# Loss of the conserved PKA sites of SIK1 and SIK2 increases sleep need

**DOI:** 10.1038/s41598-020-65647-0

**Published:** 2020-05-26

**Authors:** Minjeong Park, Chika Miyoshi, Tomoyuki Fujiyama, Miyo Kakizaki, Aya Ikkyu, Takato Honda, Jinhwan Choi, Fuyuki Asano, Seiya Mizuno, Satoru Takahashi, Masashi Yanagisawa, Hiromasa Funato

**Affiliations:** 1 International Institute for Integrative Sleep Medicine (WPI-IIIS), University of Tsukuba, Tsukuba, 305-8575 Japan; 2 Laboratory Animal Resource Center, University of Tsukuba, Tsukuba, 305-8575 Japan; 3 Department of Molecular Genetics, University of Texas Southwestern Medical Center, Dallas, TX 75390 USA; 4 Life Science Center for Survival Dynamics, Tsukuba Advanced Research Alliance (TARA), University of Tsukuba, Tsukuba, 305-8575 Ibaraki Japan; 5 Department of Anatomy, Faculty of Medicine, Toho University, Tokyo, 143-8540 Japan

**Keywords:** Molecular medicine, Physiology, Sleep

## Abstract

Although sleep is one of the most conserved behaviors, the intracellular mechanism regulating sleep/wakefulness remains unknown. We recently identified a protein kinase, SIK3, as a sleep-regulating molecule. Mice that lack a well-conserved protein kinase A (PKA) phosphorylation site, S551, showed longer non-rapid eye movement (NREM) sleep and increased NREMS delta density. S551 of SIK3 is conserved in other members of the SIK family, such as SIK1 (S577) and SIK2 (S587). Here, we examined whether the PKA phosphorylation sites of SIK1 and SIK2 are involved in sleep regulation by generating *Sik1*^*S577A*^ and *Sik2*^*S587A*^ mice. The homozygous *Sik1*^*S577A*^ mice showed a shorter wake time, longer NREMS time, and higher NREMS delta density than the wild-type mice. The heterozygous and homozygous *Sik2*^*S587A*^ mice showed increased NREMS delta density. Both the *Sik1*^*S577A*^ and *Sik2*^*S587A*^ mice exhibited proper homeostatic regulation of sleep need after sleep deprivation. Despite abundant expression of *Sik1* in the suprachiasmatic nucleus, the *Sik1*^*S577A*^ mice showed normal circadian behavior. Although *Sik2* is highly expressed in brown adipose tissue, the male and female *Sik2*^*S587A*^ mice that were fed either a chow or high-fat diet showed similar weight gain as the wild-type littermates. These results suggest that PKA-SIK signaling is involved in the regulation of sleep need.

## Introduction

Sleep is a conserved behavior in both vertebrates and invertebrates. However, the molecular mechanism regulating sleep/wakefulness remains to be elucidated. Recently, we identified a kinase, salt-inducible kinase 3 (SIK3), as an important sleep regulator through electroencephalogram (EEG)/electromyogram (EMG)-based sleep screening of randomly mutagenized mice^1^. Mice that express mutant SIK3 lacking the 52 amino acids encoded by exon 13 showed a decrease in wake time and an increase in non-rapid eye movement (NREM) sleep time. Among the 52 amino acids, serine 551 (S551) is a well-conserved protein kinase A (PKA) phosphorylation site and is crucial for the determination of sleep need^2^, suggesting that the PKA-SIK3 pathway is involved in the regulation of sleep/wakefulness.

**Figure 1:**
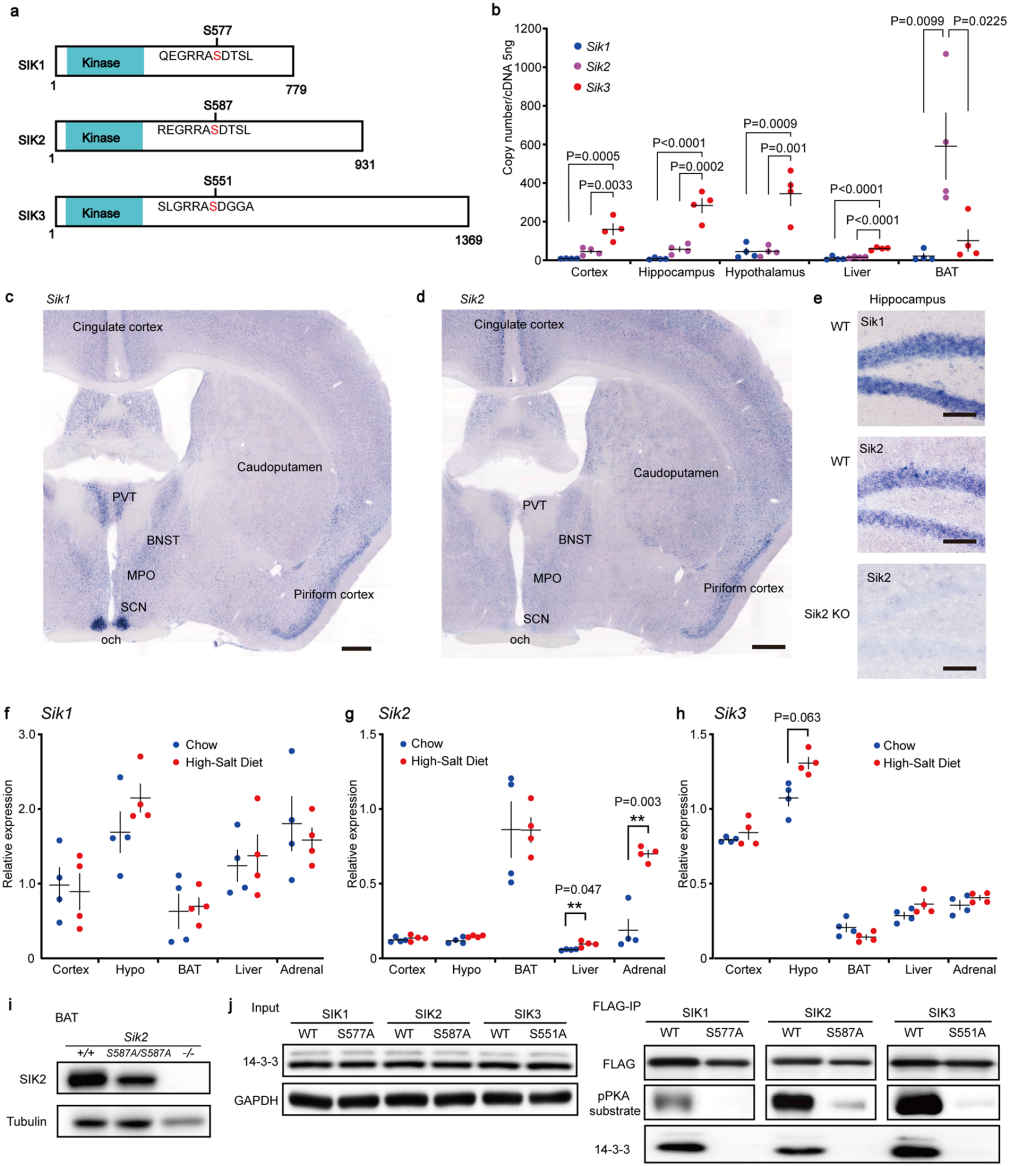
SIK family mRNA expression and mutant proteins. (**a**) Scheme of SIK1, SIK2, and SIK3. The serine residue in the PKA consensus sequence is conserved among the family. Although there are multiple protein isoforms of SIK3, this scheme shows the longest isoform. (**b**) Digital PCR results of *Sik1*, *Sik2* and *Sik3* mRNA of the cerebral cortex, hippocampus, hypothalamus, liver and brown adipose tissue (BAT) of the wild-type mice (n = 4). Each sample was measured in duplicate. One-way analysis of variance followed by Tukey’s test. (**c**-**e**) *In situ* hybridization of *Sik1* and *Sik2*. (**c**) *Sik1* mRNA was strongly expressed in the suprachiasmatic nucleus (SCN) and broadly expressed in the forebrain. Scale bar, 500 μm. (**d**) *In situ* hybridization showed that *Sik2* mRNA was broadly expressed in the forebrain. Scale bar, 500 μm. (**e**) *Sik1* and *Sik2* were expressed in the hippocampal dentate gyrus of the wild-type mice (upper and middle). *Sik2* expression was not detected in the dentate gyrus of the *Sik2*-deficient mice (lower). Scale bars, 100 μm. (**f**-**h**) *Sik1* (**f**), *Sik2* (**g**) and *Sik3* (**h**) mRNA expression in the cerebral cortex, hypothalamus, BAT, liver and adrenal gland after one week of high-salt diet feeding. Two-tailed t-test with Bonferroni correction. (i) SIK2 protein was expressed in the BAT of the *Sik2*^+/+^ and *Sik2*^*S587A/S587A*^ mice. SIK2 was not detected in the BAT of the *Sik2*-deficient mice. (j) FLAG-SIK1 WT, FLAG-SIK1 S577A, FLAG-SIK2 WT, FLAG-SIK2 S587A, FLAG-SIK3 WT, and FLAG-SIK3 S551A transiently expressed in HEK293 cells were immunoprecipitated with an anti-FLAG antibody and then subjected to immunoblotting using anti-FLAG, anti-phospho-PKA substrate motif, and anti-14-3-3 antibodies. BNST, bed nucleus of the stria terminalis; MPO, medial preoptic area; och, optic chiasma; PVT, paraventricular thalamic nucleus. One-way ANOVA followed by Tukey’s test. Data are presented as the mean ± SEM. group. Full blots are shown in the Supplementary Information.

The SIK family belongs to the AMP-activated protein kinase (AMPK) family of Ser/Thr kinases and is composed of SIK1, SIK2, and SIK3^3,4^. Similar to other members of the AMPK family, SIKs are primarily activated by LKB1 via phosphorylation of the kinase domain^5,6^. SIK1 was originally identified as an mRNA induced by a high-salt diet and was named salt-inducible kinase^7^, followed by the in silico identification of SIK2^8^ and SIK3^9^. SIK3 has three well-characterized PKA phosphorylation sites, T469, S551, and S674^10,11^. Whereas T469 of SIK3 is equivalent to SIK1 T475 and SIK2 T484, S551 of SIK3 is equivalent to SIK1 S577 and SIK2 S587^11–13^ (Fig. 1a). In addition, SIK2 has PKA phosphorylation sites, S343 and S358^11,14^. Phosphorylation of these PKA-phosphorylation sites promotes 14-3-3 binding to SIKs, which leads to changes in subcellular localization and kinase activity^2,4,10,11,14,15^. Since *Sik3*^*S551A*^ mice showed longer NREM sleep and higher NREMS delta density than wild-type mice, we focused on S551-equivalent PKA-phosphorylation sites, S577 of SIK1 and S587 of SIK2 (Fig. 1a), hypothesizing that these PKA-phosphorylation sites are involved in sleep/wake regulation similar to S551 of SIK3.

SIK1 and SIK2 have been reported to regulate circadian behavior and energy metabolism. The knockdown of SIK1 in the suprachiasmatic nucleus (SCN) resulted in a rapid phase shift of the circadian rhythm, in other words, resistance to jet lag^16^. Mice deficient in *Sik1* showed lower body weights under both chow and high-fat diets than wild-type mice^17,18^. *Sik2* is highly expressed in brown adipose tissue (BAT)^8,19^, which is a specialized thermogenic organ^20^. Whereas the *Sik2*-deficient mice showed body weights similar to those of wild-type mice^21^, mice that express SIK2 with the serine-to-alanine substitution at the 587th residue specifically in BAT were susceptible to diet-induced obesity^22^. Given that mice lacking S551 of SIK3 showed normal circadian rhythm^1^, despite altered circadian behavior of the *Sik3*-deficient mice^23^, *Sik1*^*S577A*^ and *Sik2*^*S587A*^ mice may show metabolic and circadian phenotypes that are different from those in the *Sik1*- or *Sik2*-deficient mice.

To examine whether the phosphorylation of S577 of SIK1 and S587 of SIK2 is required for proper sleep/wake behavior, we generated mutant mice in which S577 of SIK1 and S587 of SIK2 were substituted with alanine residues. Both the *Sik1* and *Sik2* mutant mice showed an increased NREMS delta, an indicator of sleep need. Consistent with the lower expression of *Sik1* and *Sik2* in the brain compared with *Sik3*, the sleep phenotypes of the *Sik1*^*S577A*^ mice and the *Sik2*^*S587A*^ mice were milder than those of the *Sik3* mutant mice. The *Sik1*^*S577A*^ mice showed normal circadian behavior and re-entrainment to a new circadian rhythm. Additionally, the male and female *Sik1*^*S577A*^ mice showed similar body weights as the wild-type littermates, and the male and female *Sik2*^*S587A*^ mice fed either a chow or high-fat diet showed a similar body weight gain as the wild-type littermates. Thus, the conserved PKA sites of SIK1 and SIK2 are thought to be required for the proper regulation of sleep need and play a minor role in circadian and body weight regulation.

## Results

### *Sik* mRNA expression in the brain and other tissues

First, we examined the mRNA levels of *Sik1, 2*, and *3* in the cerebral cortex, hippocampus, hypothalamus, liver, and BAT. *Sik*3 was the most abundant *Sik* member in the brain (Fig. 1b). *Sik1* mRNA was highly expressed in the SCN and broadly expressed in the cerebral cortex, hippocampus, thalamus, hypothalamus and brain stem (Fig. 1c,e). *Sik2* was highly abundant in the BAT as previously reported^8,19^ (Fig. 1b) and broadly expressed in the cerebral cortex, hippocampus, thalamus, and hypothalamus (Fig. 1d,e), consistent with a previous report^24^, while there was no expression in the *Sik2*-deficient mice (Fig. 1e).

Since SIK1 was originally identified as a molecule induced by a high-salt diet in the rat adrenal gland^7^, we examined whether a high-salt diet affects *Sik1, 2* and *3* expression in the brain, BAT, liver and adrenal gland. One week of a high-salt diet did not affect the *Sik1* mRNA expression in the cerebral cortex, hypothalamus, BAT, liver or adrenal gland (Fig. 1f). A high-salt diet increased the *Sik2* mRNA expression in the liver and adrenal gland (Fig. 1g) and did not cause significant changes in the *Sik3* mRNA expression in all tissues examined (Fig. 1h). We also confirmed the SIK2 protein expression in the BAT (Fig. 1i, S1a,c) and the brain (Fig. S1b,d).

### The SIK1 S577A and SIK2 S587A proteins did not bind to 14-3-3

For characterization of the SIK1 S577A and SIK2 S587A proteins, FLAG-tagged SIK protein variants were transiently expressed in HEK293 cells. Since SIKs have a RRAS motif, a consensus sequence for PKA, immunopurified FLAG-tagged SIK1, 2, and 3 were detected with an anti-phospho-PKA substrate motif antibody (Fig. 1j). In contrast, the FLAG-tagged SIK1 S577A, SIK2 S587A, and SIK3 S551A showed only faint immunoreactivity to the anti-phospho-PKA substrate motif antibody^2,10^. The 14-3-3 protein was purified with the FLAG-tagged SIK1, 2, and 3 but not with the FLAG-tagged SIK1 S577A, SIK2 S587A and SIK3 S551A (Fig. 1j), as previously reported^2,10,11^.

### The *Sik1*^*S577A*^ mice showed an increased sleep need

We generated *Sik1*^*S577A*^ mice to examine whether the presence of the PKA phosphorylation site, S577, of SIK1 is required for normal sleep/wakefulness (Fig. 1a). The external appearance, such as coat color, whisker morphology and eye shape, of the heterozygous and homozygous *Sik1*^*S577A*^ mice was indistinguishable from that of the *Sik1*^+/+^ mice. The heterozygous and homozygous *Sik1*^*S577A*^ mice did not show any abnormal locomotion or sensory response against touch or sound. The heterozygous and homozygous *Sik1*^*S577A*^ mice were fertile. The male and female *Sik1*^*S577A*^ and *Sik1*^*S577A*^ mice showed similar weights compared to the wild-type littermates at the age of 8 weeks (Fig. S1a).

**Figure 2:**
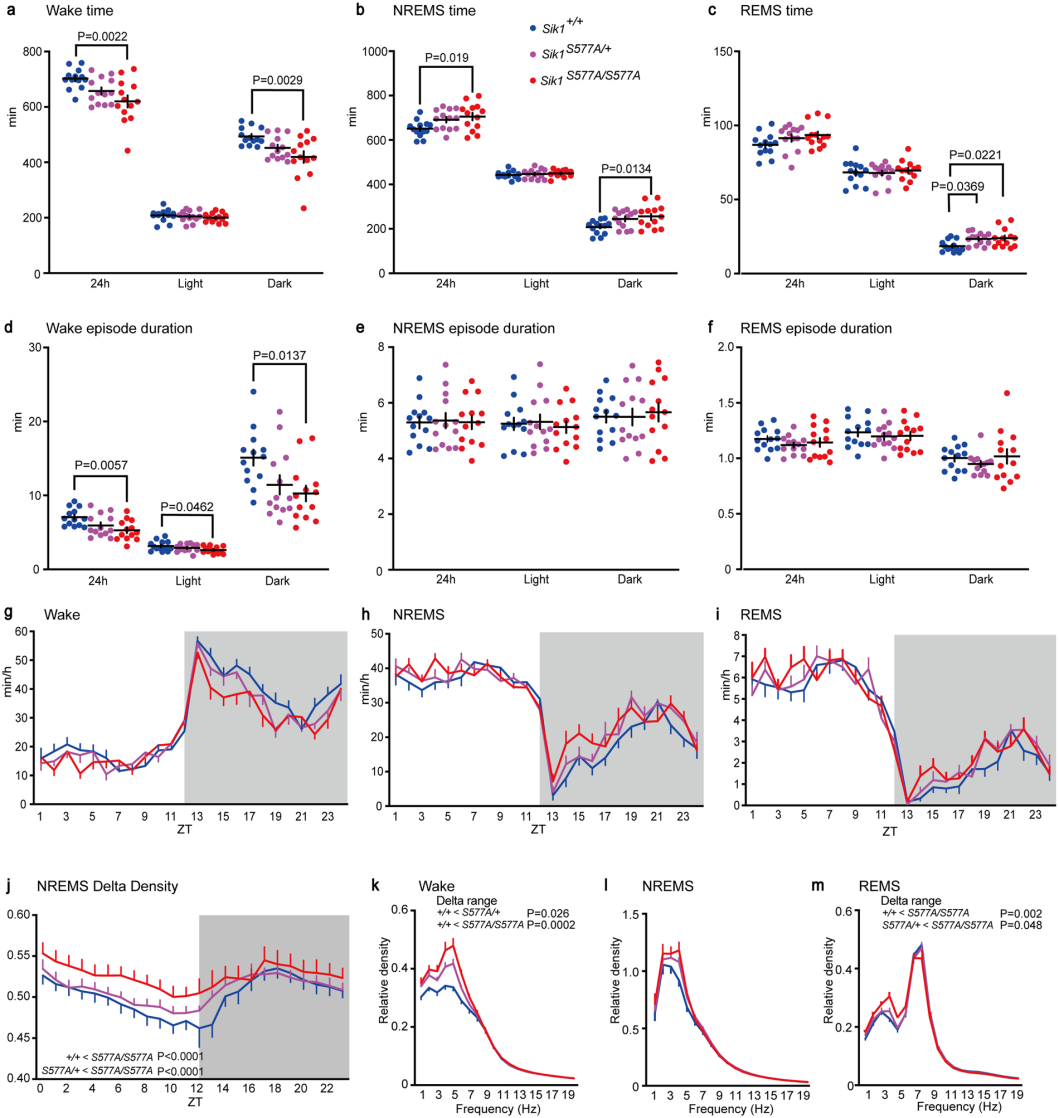
Sleep/wake behavior of the *Sik1*^*S577A*^ mice. (**a**–**c**) Total time spent in wakefulness (**a**), NREMS (**b**) and REMS (**c**) of the *Sik1*^+/+^, *Sik1*^*S577A/+*^, and *Sik1*^*S577A/S577A*^ mice. (**d**–**f**) Episode duration of wakefulness (**d**), NREMS (**e**) and REMS (**f**) of the *Sik1*^+/+^, *Sik1*^*S577A/+*^, and *Sik1*^*S577A/S577A*^ mice. (**g**–**i**) Circadian variation of wakefulness (**g**), NREMS (**h**) and REMS (**i**) of the *Sik1*^+/+^, *Sik1*^*S577A/+*^, and *Sik1*^*S577A/S577A*^ mice. (**j**) Hourly NREMS delta power density over 24 h. (**k**–**m**) EEG power density of the *Sik1*^+/+^, *Sik1*^*S577A/+*^, and *Sik1*^*S577A/S577A*^ mice during wakefulness (**k**), NREMS (**l**) and REMS (**m**). 13 male mice for each group. One-way analysis of variance (ANOVA) followed by Tukey’s test (**a**–**f**, **k**–**m**). One-way repeated-measures ANOVA followed by Tukey’s test (**j**). Data are presented as the mean ± SEM.

The *Sik1*^*S577A/S577A*^ mice exhibited a reduced total wake time over 24 h and during the dark phase (Fig. 2a) and an increased total NREMS time over 24 h and during the dark phase compared with the *Sik1*^+/+^ mice (Fig. 2b). The *Sik1*^*S577A/S577A*^ mice showed an increased total REMS time during the dark phase (Fig. 2c). There was no significant difference in the daily total time spent in any vigilance state between the *Sik1*^*S577A/+*^ and *Sik1*^+/+^ mice. For episode duration, the *Sik1*^*S577A/S577A*^ mice showed shorter wake episode durations during both the light and dark phases than the *Sik1*^+/+^ mice (Fig. 2d), whereas there were no significant changes in the NREMS episode duration (Fig. 2e) and REMS episode duration (Fig. 2f). The circadian variations of each state showed a sustained tendency toward shorter wake time and longer NREMS and REMS time during the dark phase of the *Sik1*^*S577A/S577A*^ mice (Fig. 2g–i). EEG spectral analysis revealed that the hourly NREMS delta density of the *Sik1*^*S577A/S577A*^ mice was higher than that of the *Sik1*^+/+^ and *Sik1*^*S577A/+*^ mice (Fig. 2j). The *Sik1*^*S577A/+*^ and *Sik1*^*S577A/S577A*^ mice had a higher delta range (1–4 Hz) power during wakefulness than the wild-type littermates (Fig. 2k). There was no significant difference in the delta range power during NREMS among the genotypic groups (Fig. 2l). The *Sik1*^*S577A/S577A*^ mice had a higher delta range power during REMS than the *Sik1*^*S577A/+*^ and wild-type littermates (Fig. 2m). Thus, the *Sik1*^*S577A*^ homozygous mice showed increased sleep need in terms of time and EEG spectrum.

**Figure 3:**
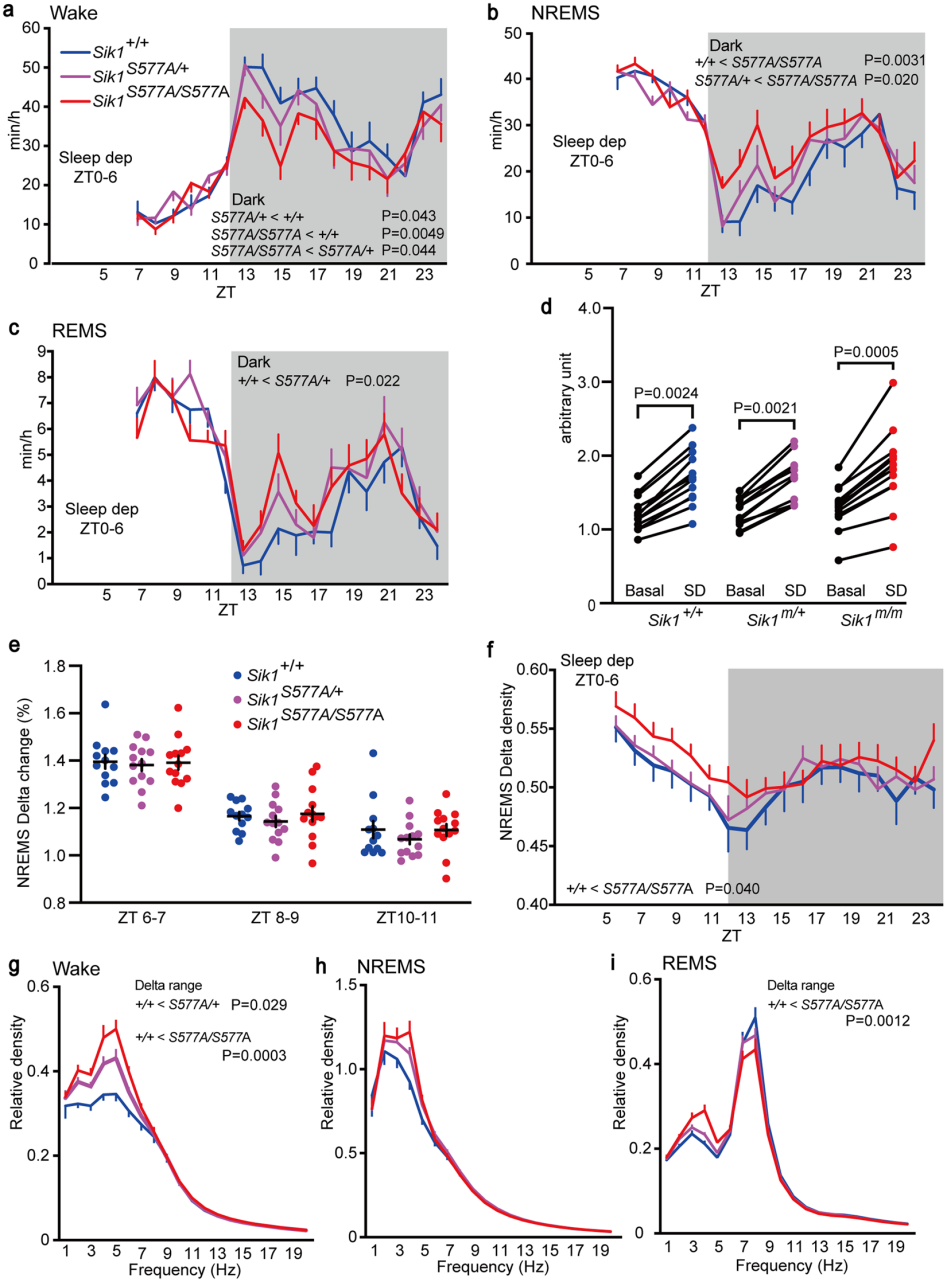
Sleep/wakefulness of the *Sik1*^*S577A*^ mice after sleep deprivation. (**a**–**c**) Hourly time spent in wakefulness (**a**), NREMS (**b**), and REMS (**c**) of the *Sik1*^+/+^, *Sik1*^*S577A/+*^, and *Sik1*^*S577A/S577A*^ mice after 6-h sleep deprivation. (**d**) NREMS delta power of the *Sik1*^*S577A*^ mice under the basal state and for two hours after 6-h sleep deprivation. (**e**) NREMS delta density change of the *Sik1*^*S577A*^ mice during ZT6-7, ZT8-9, and ZT10-11 h after 6-h sleep deprivation. (**f**) Hourly NREMS delta power density of the *Sik1*^*S577A*^ mice after 6-h sleep deprivation. (**g**–**i**) EEG power spectra of the *Sik1*^*S577A*^ mice during two hours after 6-h sleep deprivation during wakefulness (**g**), NREMS (**h**), and REMS (**i**). One-way repeated-measures analysis of variance (ANOVA) followed by Tukey’s test (**a**–**c**,**f**). Two-tailed paired T-test (**d**). Two-way ANOVA (**e**). One-way ANOVA followed by Tukey’s test (**g**–**i**). 13 male mice for each group. Data are presented as the mean ± SEM.

To investigate the homeostatic regulation of sleep/wakefulness, we performed sleep deprivation for 6 h with gentle handling. There was no difference in time spent in each vigilance state during the light phase after sleep deprivation among the genotypic groups (Fig. 3a–c). The *Sik1*^*S577A/S577A*^ mice showed a shorter wake time during the dark phase than the *Sik1*^*S577A/+*^ mice, and the *Sik1*^*S577A/+*^ mice showed a shorter wake time than the *Sik1*^+/+^ mice (Fig. 3a). Consistently, the *Sik1*^*S577A/S577A*^ mice showed longer NREMS time during the dark phase than the *Sik1*^*S577A/+*^ and *Sik1*^+/+^ mice (Fig. 3b). The *Sik1*^*S577A/+*^ mice showed longer REMS time during the dark phase than the *Sik1*^+/+^ mice (Fig. 3c). The change in NREMS delta power for 2 h after sleep deprivation was higher than that under the basal condition in all genotypic groups (Fig. 3d). Sleep deprivation increased the NREMS delta power to a similar extent among the genotypic groups (Fig. 3e). The *Sik1*^*S577A/S577A*^ mice showed a higher NREMS delta density during the light phase than the *Sik1*^+/+^ mice (Fig. 3f). Similar to basal sleep/wakefulness, the *Sik1*^*S577A/+*^ and *Sik1*^*S577A/S577A*^ mice had a higher delta power during wakefulness than the wild-type littermates after sleep deprivation (Fig. 3g), and there was no significant difference in the delta range power during NREMS among the genotypic groups (Fig. 3h). The *Sik1*^*S577A/S577A*^ mice had a higher delta range power during REMS than the wild-type littermates (Fig. 3i). These results suggest normal homeostatic sleep regulation in the *Sik1*^*S577A*^ mice.

### The *Sik2*^*S587A*^ mice showed a mild increase in sleep need

Next, we generated *Sik2*^*S587A*^ mice to investigate the role of the phosphorylation of SIK2 S587 in sleep/wake behavior (Fig. 1a). We confirmed that a SIK2 protein of the same size as wild-type protein was expressed in the homozygous *Sik2*^*S587A*^ mice (Fig. 1i). The SIK2 levels in the brains and BAT of the *Sik2*^*S587A/S587A*^ mice were lower than those of the *Sik2*^+/+^ mice (Fig. S1). The external appearance of the heterozygous and homozygous *Sik2*^*S587A*^ mice was indistinguishable from that of the *Sik2*^+/+^ mice. Although there was no significant difference in body weight at the age of 8 weeks of the male mice with the *Sik2*^*S587A*^ genotype (Fig. S2b), the body weight of the female heterozygous and homozygous *Sik2*^*S587A*^ mice was lower than that of the wild-type littermates. The heterozygous and homozygous *Sik2*^*S587A*^ mice did not show any abnormal locomotion or sensory response. The heterozygous and homozygous *Sik2*^*S587A*^ mice were fertile.

**Figure 4:**
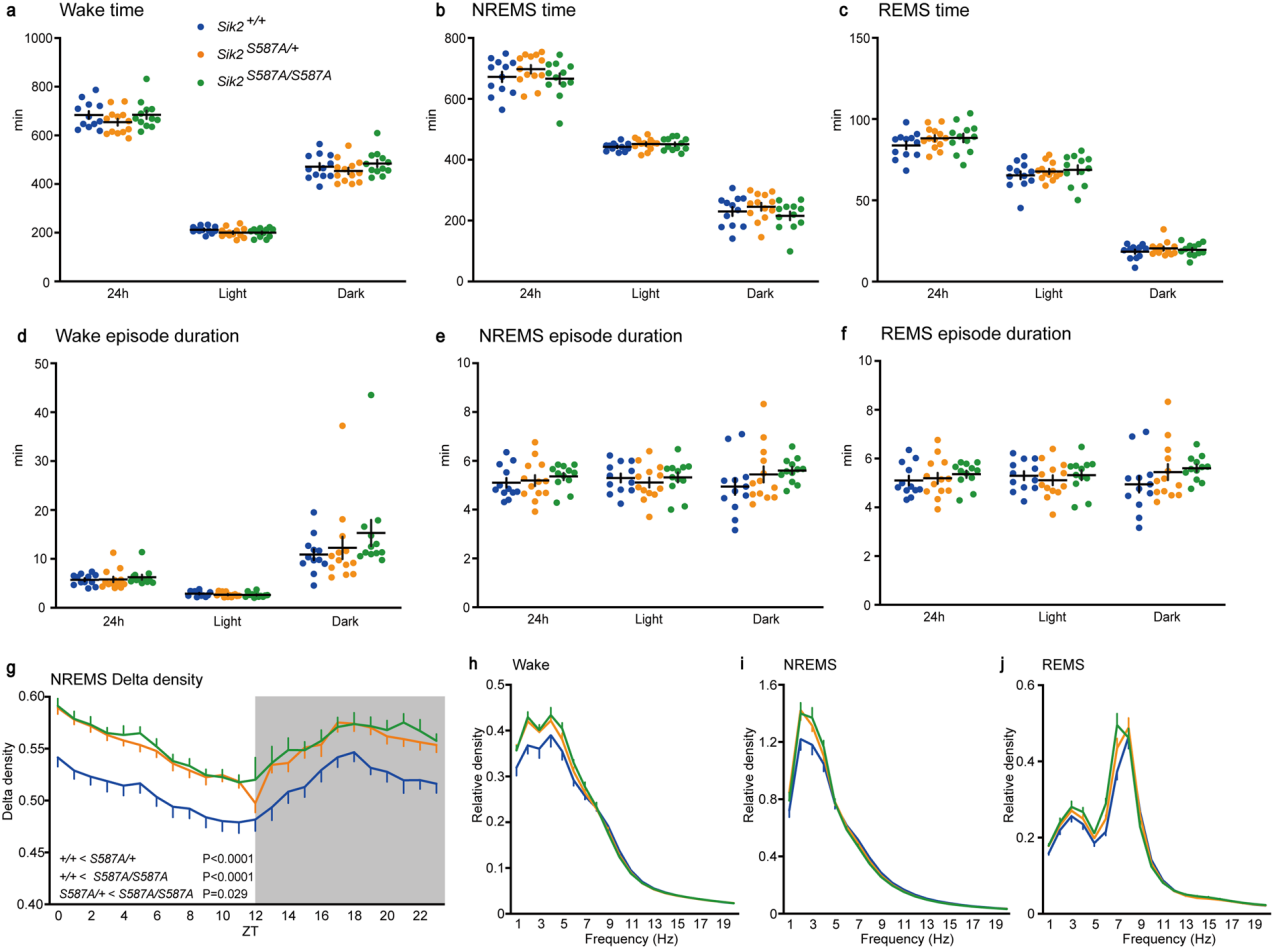
Sleep/wake behavior of the *Sik2*^*S587A*^ mice. (**a**–**c**) Total time spent in wakefulness (**a**), NREMS (**b**) and REMS (**c**) of the male *Sik2*^+/+^, *Sik2*^*S587A/+*^, and *Sik2*^*S587A/S587A*^ mice. (**e**–**f**) Episode duration of wakefulness (**d**), NREMS (**e**) and REMS (**f**) of the *Sik2*^+/+^, *Sik2*^*S587A/+*^, and *Sik2*^*S587A/S587A*^ mice. (**g**) Hourly NREMS delta power density over 24 h. (**h**–**j**) EEG power density of the *Sik2*^+/+^, *Sik2*^*S587A/+*^, and *Sik2*^*S587A/S587A*^ mice during wakefulness (**h**), NREMS (**i**) and REMS (**j**). *Sik2*^+/+^, n = 12; *Sik2*^*S587A/+*^, n = 13; *Sik2*^*S587A/S587A*^, n = 12. One-way analysis of variance (ANOVA) followed by Tukey’s test (**a**–**f**, **h**–**j**). One-way repeated-measures ANOVA followed by Tukey’s test (**g**). Data are presented as the mean ± SEM.

Both the *Sik2*^*S587A/+*^ and *Sik2*^*S587A/S587A*^ mice had a total time spent in each vigilance state similar to that of the wild-type mice (Fig. 4a–c). There was no significant difference in episode duration of each vigilance state among the genotypic groups (Fig. 4d–f). EEG spectral analysis revealed a higher NREMS delta density of the *Sik2*^*S587A/+*^ and *Sik2*^*S587A/S587A*^ mice than that of the *Sik2*^+/+^ mice throughout the entire day (Fig. 4g). There was no difference in the delta range power in each vigilance state among the genotypic groups (Fig. 4h–j). Thus, the *Sik2*^*S8587A*^ homozygous mice showed increased sleep need in terms of EEG spectrum.

**Figure 5:**
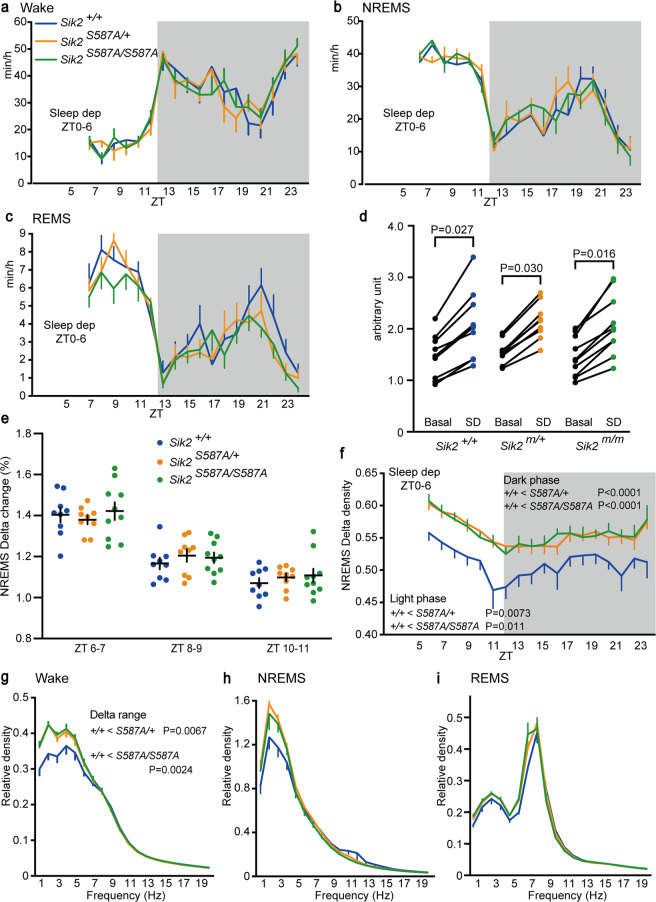
Sleep/wakefulness of the *Sik2*^*S587A*^ mice after sleep deprivation. (**a**–**c**) Hourly time spent in wakefulness (**a**), NREMS (**b**), REMS (**c**) of the male *Sik2*^+/+^, *Sik2*^*S587A/+*^, and *Sik2*^*S587A/S587A*^ mice. (**d**) NREMS delta power of the *Sik2*^*S587A*^ mice under the basal state and for 2 h after 6-h sleep deprivation. (**e**) NREMS delta density change during ZT6-7, ZT8-9, and ZT10-11 h after 6-h sleep deprivation. (**f**) Hourly NREMS delta power density of the *Sik2*^*S587A*^ mice after 6-h sleep deprivation. (**g**-**i**) EEG power spectra of the *Sik2*^*S587A*^ mice during two hours after 6-h sleep deprivation during wakefulness (**g**), NREMS (**h**), REMS (**i**). *Sik2*^+/+^, n = 9; *Sik2*^*S587A/+*^, n = 9; *Sik2*^*S587A/S587A*^, n = 10. One-way repeated-measures analysis of variance (ANOVA) followed by Tukey’s test (**a**–**c**, **f**). Two-tailed paired T-test (**d**). Two-way ANOVA (**e**). One-way ANOVA followed by Tukey’s test (**g**–**i**). Data are presented as the mean ± SEM.

After sleep deprivation for 6 h, the amounts of time spent in wakefulness, NREMS and REMS were similar among the genotypic groups during the light and dark phases (Fig. 5a–c). The change in NREMS delta power over 2 h after sleep deprivation was higher than that under the basal condition in all genotypic groups (Fig. 5d). Sleep deprivation increased the NREMS delta power to a similar extent among the genotypic groups (Fig. 5e). Similar to basal sleep/wakefulness, the *Sik2*^*S587A/+*^ and *Sik2*^*S587A/S587A*^ mice showed a higher NREMS delta density during the light and dark phases than the *Sik2*^+/+^ mice (Fig. 5f). The *Sik2*^*S587A/+*^ and *Sik2*^*S587A/S587A*^ mice showed higher delta range power during wakefulness than the *Sik2*^+/+^ mice (Fig. 5g). There was no difference in the delta range power in NREMS and REMS among the genotypic groups (Fig. 5h,i).

### Normal circadian behavior of the *Sik1*^*S577A*^ mice

**Figure 6:**
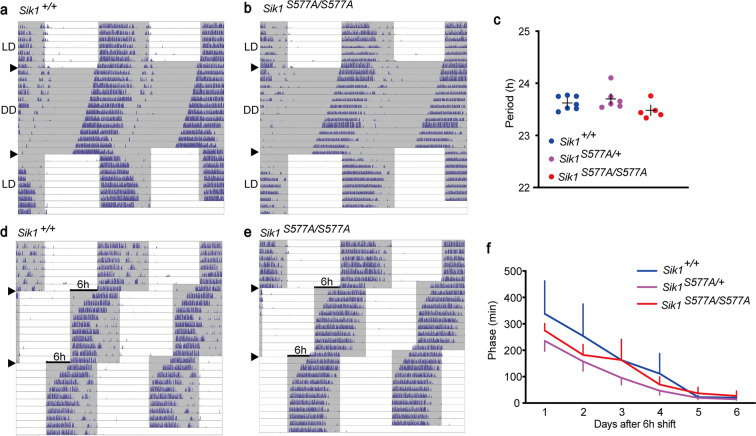
Circadian behavior of the *Sik1*^*S577A*^ mice. (**a**,**b**) Double-plot of wheel-running activity under constant darkness (DD) in the male *Sik1*^+/+^ (**a**) and *Sik1*^*S577A/S577A*^ mice (**b**). (**c**) Circadian period of the *Sik1*^*S577A*^ mice. One-way analysis of variance (ANOVA). (**d**,**e**) The LD cycle was advanced by 6 hours twice every 10 days. (**f**) Phase relative to the new LD cycle (first shift) of the *Sik1*^*S577A*^ mice. Data plotted against days after the shift in cycle. n = 7 for *Sik1*^+/+^ mice, n = 6 for *Sik1*^*S577A/+*^ mice and n = 5 for *Sik1*^*S577A/S577A*^ mice. One-way repeated-measures ANOVA. Data are presented as the mean ± SEM.

Since *Sik1* is expressed in the SCN (Fig. 1c) and was reported to be involved in the phase shift of circadian rhythm based on deficiency studies^16^, we examined the circadian behavior of the *Sik1*^*S577A*^ mice. Under constant darkness, the *Sik1*^*S577A/+*^ and *Sik1*^*S577A/S577A*^ mice showed a circadian period similar to that of the wild-type littermates (Fig. 6a–c). Furthermore, the *Sik1*^*S577A/+*^ and *Sik1*^*S577A/S577A*^ mice showed re-entrainment to a new circadian time with a similar phase shift to the wild-type mice (Fig. 6d–f). Thus, *Sik1*^*S577A*^ mutant mice showed normal circadian behavior in terms of endogenous circadian period length and re-entrainment.

### Normal body weight growth of the *Sik2*^*S587A*^ mice

**Figure 7:**
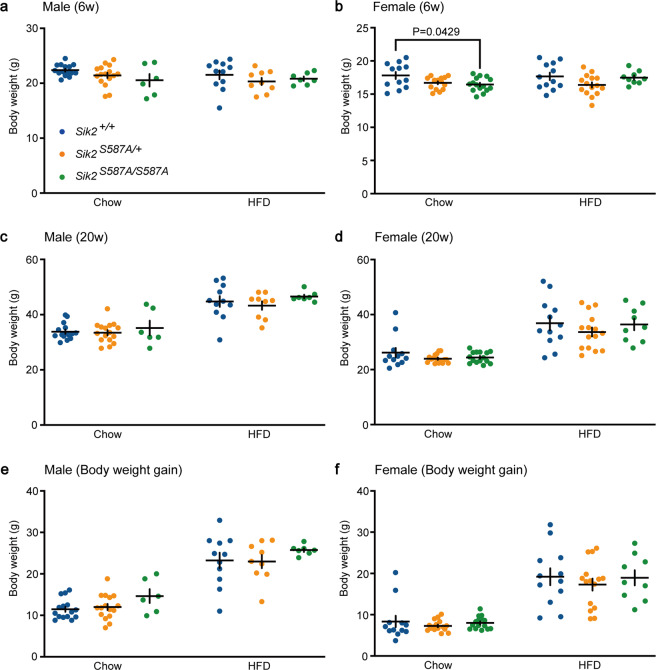
Body weight growth of the *Sik2*^*S587A*^ mice fed chow or a high-fat diet. (**a**) Body weights of the male *Sik2*^*S587A*^ mice at the age of 6 weeks. (**b**) Body weights of the female *Sik2*^*S587A*^ mice at the age of 6 weeks. (**c**) Body weights of the male *Sik2*^*S587A*^ mice at the age of 20 weeks. (**d**) Body weights of the female *Sik2*^*S587A*^ mice at the age of 20 weeks. (**e**) Body weight gain of the male *Sik2*^*S587A*^ mice from 6 weeks to 20 weeks of age. (**f**) Body weight gain of the female *Sik2*^*S587A*^ mice from 6 weeks to 20 weeks of age. Two-way analysis of variance followed by Sidak’s test. All data are presented as the mean ± SEM. Male *Sik2*^+/+^ mice (chow n = 15, HFD n = 11). Male *Sik2*^*S587A/+*^ mice (chow n = 16, HFD n = 9). Male *Sik2*^*S587A/S587A*^ mice (chow n = 6, HFD n = 7). Female *Sik2*^+/+^ mice (chow n = 12, HFD n = 12). Female *Sik2*^*S587A/+*^ mice (chow n = 16, HFD n = 15). Female *Sik2*^*S587A/S587A*^ mice (chow n = 15, HFD n = 9).

Since *Sik2* is highly expressed in BAT (Fig. 1b), which regulates energy metabolism, we examined the body weight growth of the *Sik2*^*S587A*^ mice. At the age of 6 weeks, the body weights of the male *Sik2*^*S587A*^ mice fed a chow or a high-fat diet were similar to those of the wild-type littermates (Fig. 7a). The body weights of the female *Sik2*^*S587A/S587A*^ mice on chow were smaller than those of the *Sik2*^+/+^ mice (Fig. 7b). However, there was no difference between the female *Sik2*^+/+^ and *Sik2*^*S587A/S587A*^ mice on a high-fat diet (Fig. 7b). At the age of 20 weeks, both male and female *Sik2*^*S587A*^ mice fed a chow or a high-fat diet showed body weights similar to those of their wild-type littermates (Fig. 7c,d). There was no significant difference in the body weight gain from 6 weeks to 20 weeks of age among the genotypic groups for both sexes (Fig. 7e,f). Thus, male and female *Sik2*^*S587A*^ mice have normal body weight regulation.

## Discussion

In this study, we examined the sleep/wake behavior of mice lacking conserved PKA phosphorylation sites of SIK1 and SIK2. Both the *Sik1*^*S577A*^ mice and the *Sik2*^*S587A*^ mice showed an increased NREMS delta density, an index of sleep, which is consistent with the results of the *Sik3*^*Slp*^ mice that lack an exon 13-encoded region and the *Sik3*^*S551A*^ mice that lack a conserved PKA phosphorylation site^1,2^.

The homozygous *Sik1*^*S577A*^ mice showed a shorter total wake time and longer total NREMS time than the wild-type mice in addition to an increased NREMS delta density. Thus, the *Sik1*^*S577A*^ mice had a sleep phenotype similar to that of the *Sik3* mutant but to a lesser degree, which is explained by the lower *Sik1* expression than *Sik3* expression in the brain. Increased delta power during wakefulness and REMS of the *Sik1*^*S577A*^ mice may indicate sleep need during wakefulness and REMS, which was also observed in the *Sik3*^*Slp*^ mice^1^. The sleep-deprived *Sik1*^*S577A*^ mice showed a longer NREMS time and higher NREMS delta density than the sleep-deprived wild-type littermates. Sleep deprivation increased the NREMS delta density to a similar extent, suggesting that the *Sik1*^*S577A*^ mice showed proper homeostatic sleep regulation.

Both the heterozygous and homozygous *Sik2*^*S587A*^ mice exhibited an increased NREMS delta density, but the *Sik2*^*S587A*^ mice did not alter the time spent in wakefulness and sleep. Lower brain expression of *Sik2* than *Sik3* accounts for the milder sleep phenotype of the *Sik2*^*S587A*^ mice than that of the *Sik3*^*Slp*^ and *Sik3*^*S551A*^ mice. Differential expression patterns between *Sik2* and *Sik3* may cause normal sleep time of the *Sik2*^*S587A*^ mice. Thus, the current results as a whole support the idea that PKA-SIK signaling is involved in the regulation of sleep need. Importantly, KIN-29, the *Caenorhabditis elegans* homologue of SIK is involved in the regulation of sleep^1,25^.

Consistent with previous studies^2,10,11^, the SIK1 S577A, SIK2 S587A, and SIK3 S551A proteins showed reduced immunoreactivities for phospho-PKA substrate motifs. The remaining immunoreactivities for the phospho-PKA motif of SIK1 S577A, SIK2 S587A, and SIK3 S551A are attributed to other PKA recognition sites, such as SIK1 T475, SIK2 T484 and SIK3 T469^11^. In addition, the SIK1 S577A, SIK2 S587A, and SIK3 S551A proteins showed decreased binding to 14-3-3, as previously reported^2,10,11^. Importantly, each PKA phosphorylation site of SIKs has differential effects on binding to 14-3-3. Berggreen *et al*. showed much lower 14-3-3 binding of the SIK3 T469A and SIK3 S551A proteins than the wild-type SIK3 using HEK293 cells^10^. Sonntag *et al*. showed that the alanine substitution of SIK2 at S358 or S587 led to a similar decrease in 14-3-3 binding in HEK 293 cells^11^, which is consistent with the decreased 14-3-3 binding observed in the present study. However, Henriksson *et al*. showed that the alanine substitution of S343 or S358 of SIK2 decreased 14-3-3 binding, but the alanine substitution of SIK2 S587 did not affect binding to 14-3-3 in HEK293 cells^14^. Although the reason for this discrepancy is unknown, the effect of PKA on SIKs may be omplex depending on the context and the presence of other upstream kinases^4^.

The phosphorylation of the PKA site of SIK1 regulates the nucleo-cytoplasmic localization of SIK1^6,8,13^, whereas SIK2 and SIK3 remain localized in the cytoplasm^10,11,13,19^. Regardless of subcellular localization, it was reported that SIKs commonly phosphorylate the same substrates. cAMP-responsive element-binding protein (CREB)-regulated transcription coactivators (CRTCs) are the best characterized substrates of SIKs^4,12,26,27^. Class IIA histone deacetylases (HDACs) have also been reported as SIK substrates^4,28–30^. Phosphorylation of SIKs by PKA prevents SIKs from phosphorylating substrates through 14-3-3 binding and/or nucleocytoplasmic translocation^4,10,11,15^, which subsequently alters gene expression that may lead to altered phosphorylation of sleep need-index phosphoproteins (SNIPPs)^31^.

Consistent with a previous report indicating that SIK1 in the SCN is necessary for proper circadian re-entrainment^16^, SIK1 is highly expressed in the SCN. However, the *Sik1*^*S577A*^ mice showed normal circadian rhythm and re-entrainment to an advanced light-dark cycle, suggesting that the functional change of SIK1 has little effect on circadian behavior. Similarly, the *Sik3*^*Slp*^ mice showed an enhanced sleep need and normal circadian behavior^1^, while SIK3 deficiency caused abnormal circadian behavior in mice and fruit flies^23,32^. However, since systemic SIK3 deficiency caused severe growth retardation and metabolic and skeletal abnormalities^33,34^, neuron-specific SIK3 deficiency is necessary to examine the role of brain SIK3 in circadian behavior in detail.

Male *Sik2*^*S587A*^ mice exhibited body weights similar to those of the wild-type littermates fed either a high-fat diet or chow. Although the female *Sik2*^*S587A*^ mice showed lower body weights than the wild-type controls at the ages of 6 and 8 weeks, they showed a normal body weight gain as they grew. These results are not consistent with a previous report demonstrating that mice with specific *Sik2*^*S587A*^ overexpression in BAT showed high-fat diet-induced obesity^22^. Thus, the SIK2 S587A protein in the BAT may promote weight gain, but SIK2 S587A outside the BAT may counteract the effect on the BAT. Although we did not examine this issue in our study, the *Sik2*^*S587A*^ mice may show altered glucose metabolism since higher blood glucose was found in *Sik2*-deficient mice^35^ and those with pancreatic beta cell specific *Sik2* ablation^36^, but not liver-specific SIK2 deficiency, than wild-type mice^19^. Although the mice deficient in *Sik1* showed lower body weights under both chow and high-fat diets^17,18^, the male and female *Sik1*^*S577A*^ mice did not show any significant change in body weight. Thus, PKA-SIK1/2 signaling does not play a major role in energy metabolism.

We also examined whether a high-salt diet affects *Sik* expression. Although a high-salt diet for seven days strongly induced *Sik1* mRNA in the adrenal gland of rats^7^, we did not observe a significant increase in *Sik1* mRNA in the adrenal gland of mice. This unexpected result may be due to species differences and/or some technical differences. Instead, we found that a high-salt diet increased the *Sik2* mRNA levels in the adrenal gland and liver. *Sik1* mRNA showed a higher individual difference than *Sik2* and *Sik3* mRNA.

One limitation of our study is the protein level characterization of mutant mice. Although several antibodies for SIK1 are commercially available, all the SIK1 antibodies we tested did not work. Therefore, we generated polyclonal antibodies in rabbits for SIK1. However, our SIK1 antibodies are not sensitive enough to detect endogenous SIK1, which may be expressed at a low level according to the digital PCR results. For SIK2, a good anti-SIK2 antibody (Cell Signaling Technology #6919) detected SIK2 in the BAT of the wild-type mice, and the corresponding bands were not detected in the SIK2-deficient mice (Fig. S4a). However, this SIK2 antibody showed a band similar in size to SIK2 in brains, which was found for both the wild-type and SIK2 KO mice (Fig. S4b). Then, we used a different antibody (Merck #07-1378), which detects SIK2 in brains (Fig. S4d) and BAT (Fig. S4c). Although identification of endogenous proteins interacting with SIKs in the brain is crucial for the mechanistic understanding of how the SIK family regulates sleep need, the low abundance of SIK2 protein in the brain makes immunoprecipitation difficult. This issue needs to be addressed in future studies using mice that have endogenous SIKs with epitope tags.

We found that SIK2 (S587A) protein level in the brains of the *Sik2*^*S587A/S587A*^ mice was lower than the level of SIK2 protein in the wild-type mice. Additionally, the amount of SIK2 (S587A) protein in BAT of the *Sik2*^*S587A/S587A*^ mice was lower than the amount of SIK2 protein in the wild-type mice. This result suggests that phosphorylation at S587A affects the protein amount and/or stability. Since 14-3-3 has been reported to stabilize the binding protein^37,38^, reduced binding of SIK2 S587A to 14-3-3 may result in reduced SIK2 S587A levels. Thus, PKA may regulate SIK2 in multiple aspects, including kinase activity and protein stability, via 14-3-3 binding, which leads to altered phosphorylation of substrates and sleep need.

In summary, the current study broadened and confirmed the hypothesis that PKA-SIK signaling constitutes an intracellular signaling pathway regulating sleep/wakefulness, as was originally conceived based on findings in *Sik3* mutant mice. Elucidation of downstream signals is a challenge for the near future.

## Methods

### Animals

All animal experiments were conducted in accordance with the Guidelines for Animal Experiments of the University of Tsukuba and were approved by the Institutional Animal Care and Use Committee of the University of Tsukuba (approved protocol ID #180094). *Sik1*^*S577A*^ and *Sik2*^*S587A*^ mice and littermate wild-type mice were maintained on the C57BL/6 background. Male C57BL/6 mice were obtained from CLEA Japan. *Sik2*-deficient mice^21,39^ (#nbio071) were obtained from the Animal Resource Bank of National Institutes of Biomedical Innovation, Health and Nutrition, Japan. Mice were housed under humidity- and temperature-controlled conditions on a 12:12 h light/dark cycle. Food was provided ad libitum. Mice were weaned at 4 weeks of age and were housed in groups of four or five.

### Generation of the *Sik1*^*S577A*^ and *Sik2*^*S587A*^ mice

We created *Sik1*^*S577A*^ and *Sik2*^*S587A*^ mice as previously reported with some modifications^40^. In brief, to produce the *Sik1*^*S577A*^ and *Sik2*^*S587A*^ mice, we used Cas9 protein, guide RNA (gRNA) and donor oligonucleotide. The gRNAs synthesized using the GeneArt Precision gRNA Synthesis Kit (ThermoFisher), were 5′-TCTGATACCTCTCTCACTCA-3′ for *Sik1*^*S577A*^ and 5′-TCAGATACGTCCCTTACACA-3′ for *Sik2*^*S587A*^. The donor oligonucleotides for *Sik1* and *Sik2* were 151- and 113-bases (Integrated DNA Technologies), respectively, which have a sequence to introduce a serine-to-alanine substitution and several silent mutations to disrupt the PAM sequence. Pregnant mare serum gonadotropin and human chorionic gonadotropin were intraperitoneally injected into female C57BL/6 N mice (Charles River Laboratories), and unfertilized oocytes were collected from their oviducts. We then performed *in vitro* fertilization with these oocytes and sperm from male C57BL/6 N mice. The gRNA, donor oligonucleotide, and GeneArt Platinum Cas9 Nuclease (ThermoFisher) were electroporated into zygotes using a NEPA 21 electroporator (NEPAGNENE). After electroporation, embryos developed to the morula stage in KSOM-AA medium. The next day, the electroporated embryos, which developed to the two-cell stage, were transferred into pseudopregnant ICR mice. The genomic DNA sequence of F0 mice was examined by direct sequencing. For routine genotyping, we used the dCAPS method^41^ using primers designed with dCAPS Finder 2.0 (http://helix.wustl.edu/dcaps/dcaps.html). For *Sik1*^*S577A*^ genotyping, the genomic DNA was amplified using *Sik1-Fw* (5′-TCTGG GCACAGCCGTCTTA-3′) and *Sik1-Rv* (5′-CTGCACAGGCTGGGATGA-3′) to produce a 190-bp fragment and then digested with DdeI to produce 70-bp and 120-bp fragments when the allele was wild-type. For *Sik2*^*S587A*^ genotyping, the genomic DNA was amplified using *Sik2-Fw* (5′-TCCCCCGTGAGCTTCCGAGAAGGCCGCAGAGAA-3′) and *Sik2-Rv* (5′-CCTTTGGTTCTAGCAAGATTCTG-3′) to produce a 215-bp fragment and then digested with Hinf1 to produce 180-bp and 30-bp fragments when the allele was wild-type. F0 mice were mated with wild-type C57BL/6 mice to obtain heterozygous F1 offspring. Behavioral analysis was performed using F1 or later generations. Appearance, basal motor, and sensory responses were examined according to modified SHIRPA^42^.

### High-salt diet feeding

For a high-salt diet, NaCl was added to MF (containing 0.3% NaCl, Oriental Yeast) to a concentration of 6.6% NaCl by weight^43–45^, and then water was added to mix NaCl and MF. Mice were fed a high-salt diet or an MF chow diet for one week. One week of feeding with a high-salt diet did not affect the body weight but increased water intake and urination.

### EEG/EMG electrode implantation surgery

At 8–12 weeks of age, male mice were implanted with EEG/EMG electrodes with 4 EEG electrode pins and 2 flexible stainless EMG wires under anesthesia using isoflurane (4% for induction, 2.5% for maintenance)^46^. The EEG electrode pins were positioned over the frontal and occipital cortices (anteroposterior (AP): 0.5 mm, mediolateral (ML): 1.3 mm, dorsoventral (DV): − 1.3 mm and AP: − 4.5 mm, ML: 1.3 mm, DV: − 1.3 mm) and attached to the skull using dental cement. Then, the EMG wires were inserted into the neck muscles. All mice were allowed at least 4 to 7 days for recovery from the surgery. After the recovery period, all mice were attached to a tether cable and then allowed to habituate to the recording conditions for 7 days.

### EEG/EMG recording and analysis

EEG/EMG recordings were analyzed as previously described^46^. The recording room was kept under a 12:12 h light/dark cycle and a constant temperature (24–25 °C). The EEG/EMG data were visualized and semiautomatically analyzed by MATLAB-based software. The sleep/wake state in each 20-s epoch was classified as NREMS, REMS or wakefulness. Wakefulness was scored based on the criteria of the presence of fast EEG, high amplitude and variable EMG. NREMS was staged based on high amplitude, delta (1–4 Hz) frequency EEG and low EMG tonus. REMS was characterized by theta (6–9 Hz)-dominant EEG and EMG atonia. For analysis of baseline sleep/wake behavior, EEG/EMG signals were recorded for 2 consecutive days from the onset of the light phase, ZT0. The total amounts of time spent in wakefulness, NREMS and REMS were derived by summing the total number of 20-s epochs in each state. Mean episode durations were determined by dividing the total time spent in each state by the number of episodes of that state. EEG signals were subjected to fast Fourier transform analysis from 1 to 30 Hz with 1–Hz bins using MATLAB-based custom software. For normalization of the EEG power density, the EEG power density in each frequency bin was expressed as a percentage of the mean EEG power in a relatively constant higher frequency range (16–30 Hz)^2^. The hourly delta density during NREMS indicates the hourly averages of delta density, which is the ratio of delta power (1–4 Hz) to total EEG power (1–30 Hz) at each 20-s epoch or all epochs. For sleep deprivation, the mice were sleep-deprived for six hours from the onset of light phase ZT0 by gentle handling^47^. During that time, food and water were available. For evaluation of the effect of sleep deprivation, NREMS delta power during the first hour after sleep deprivation was expressed relative to the same ZT of the basal recording or relative to the mean of the basal recording.

### *In situ* hybridization

*In situ* hybridization was performed as previously described with some modifications^48^. Fragments of the coding regions for *Sik1* and *Sik2* 0.7–0.8 kb in length were generated by PCR using mouse brain and BAT cDNA as templates, respectively. The primers used were *Sik1-Fw* (5′-ATAGA CTGTG ATCTC CACAG CTCAC TT-3′), *Sik1-Rv* (5′-ACAGG GAGCA AGCAC ATAGG-3′), *Sik2-Fw* (5′-AACCC CTCCC TTGAG AGTGT-3′) and *Sik2-Rv* (5′-GGAAG AGTCG CTTCT GTTGG-3′). *Sik1* and *Sik2* cDNAs were inserted into pGEM-T easy (Promega) and used for digoxigenin (DIG)-labeled probe synthesis. Mice were perfused with PBS followed by 4% paraformaldehyde (PFA), and harvested brains were postfixed in 4% PFA overnight. Forty μm-thick brain sections were treated with 0.3% Triton X-100, digested with 1 μg/ml proteinase K, treated with 0.75% glycine, and then treated with 0.25% acetic anhydride in 0.1 M triethanolamine. After overnight incubation with a DIG-labeled probe at 60 °C, the sections were washed and then incubated with alkaline phosphatase-conjugated anti-DIG Fab fragments (Roche). The reactions were visualized with a 5-bromo-4-chloro-3-indolyl-phosphate/4-nitroblue tetrazolium (BCIP/NBT) substrate solution (Roche). *Sik2-*deficient mouse brains were used as negative controls^39^.

### Quantitative RT-PCR and digital PCR

RNA was prepared from the mouse brain, liver, BAT and adrenal gland using the RNeasy Lipid Tissue Mini Kit (Qiagen) and QIAzol Lysis Reagent (Qiagen). cDNA was synthesized using oligo dT primer and RNAs as a template with the PrimeScript Reverse Transcriptase Kit (TaKaRa). To quantitate *Sik* mRNA in each tissue, we used the QX200 Droplet Digital PCR system (Bio-Rad). We prepared a mixture containing cDNA, primers, and ddPCR EvaGreen (Bio-Rad), and processed the mixture for the QX200 Droplet Generator to create droplets. Then, we performed digital PCR and read duplicated droplets using a QX200 Reader according to the manufacturer’s instructions.

For quantitation of the effect of a high-salt diet, cDNAs were subjected to real-time PCR (ViiA7; ThermoFisher) using SYBR PreMix Ex Taq (TaKaRa) and gene-specific primers. The expression of *Sik1, 2*, and 3 in each tissue, was normalized to the *glyceraldehyde-3-phosphate dehydrogenase* (*Gapdh*) gene^49^. The primers used were *Gapdh-Fw* (5′-AGAAC ATCAT CCCTG CATCC-3′), *Gapdh-Rv* (5′-CACAT TGGGG GTAGG AACAC-3′), *Sik1-Fw* (5′-GACGG AGAGC GTCTG ATACC-3′), *Sik1-Rv* (5′-GAGCC AACCC TTTGA TCTTG-3′), *Sik2-Fw* (5′-TCCAA GACCT TTCGA GCAGT-3′), *Sik2-Rv* (5′-CAAGC TTTGC TGTGG TGAGA-3′), *Sik3-Fw* (5′-CCAGA GCTCT TCGAA GGGAA-3′) and *Sik3-Rv* (5′-CGTTT GATGG GAGCA CACTG-3′).

### High-fat diet and body weight

Body weight was measured weekly from the age of 6 weeks to 20 weeks. At 6 weeks of age, mice were assigned to a chow diet (MF; Oriental Yeast) or a high-fat diet (D12492; Research Diets). The normal chow diet (MF; Oriental Yeast) provided 3.6 kcal/g (61% carbohydrate, 26% protein, and 13% fat), and the high-fat diet provided 5.2 kcal/g (20% carbohydrate, 20% protein, and 60% fat).

### Circadian behavior

Male mice were housed individually in a cage containing a wireless running wheel (Med Associate #ENV-047). The rotation numbers of the wheels were collected by Wheel Manager software (Med Associate) via a receiving device (Med Associate #DIG807). The mice were entrained on the running wheels on a 12 h: 12 h light-dark cycle for 7 days and then allowed to run freely for 14 days under constant darkness. For analysis of re-entrainment to a new light-dark cycle, the mice were maintained on a 12 h: 12 h light-dark cycle, and then, the cycle was advanced by six hours. Ten days later, the cycle was advanced again by six hours. The circadian period and re-entrainment were analyzed by a Python-based program. The onset of activity on each day was used to measure the phase relative to the light-dark cycle^16^. The free-running period was calculated with linear regression analysis of activity onset using a Python-based program. The circadian activity amplitude was normalized to the mean amplitude of the wild-type group^1^.

### Cell culture and plasmids for transfection

HEK293 cells (RCB2202) were obtained from the RIKEN BRC Cell Bank. HEK293 cells were cultured and passaged under 5% CO_2_ in DMEM containing 10% fetal bovine serum and penicillin/streptomycin. Then, 3xFLAG-tagged mouse *Sik1*, mouse *Sik2*, and mouse *Sik3* cDNAs were cloned into the pcDNA3.1 vector using an In-Fusion HD Cloning Kit (TaKaRa). Although there are multiple protein isoforms of SIK3, we found that SIK3 isoform ending at exon 14 (e.g. UniProt: Q6P4S6-2) is a major form (Ikkyu *et al*., unpublished data). We used this SIk3 isoform in this study. Expression vectors for FLAG-SIK1 (S577A) and FLAG-SIK2 (S587A) were generated from pcDNA3-FLAG-SIK1 and pcDNA3-FLAG-SIK2 using a KOD-Plus-Mutagenesis Kit (Toyobo) and a PrimeSTAR Mutagenesis Basal Kit (TaKaRa). Cells were grown to 80% confluency in 6-well plates. HEK293 cells were transfected with 3 μg of pcDNA for FLAG-SIK1, FLAG-SIK1 S577A, FLAG-SIK2, FLAG-SIK2 S587A, 3xFLAG-SIK3, and 3xFLAG-SIK3 S551A by using 9 μl of FuGENE HD Transfection Regent (Promega).

### Immunoprecipitation and western blot

Used primary antibodies were anti-DYKDDDDK tag antibody (mouse, Monoclonal, Clone #1E6, Wako) as anti-FLAG antibody, anti-phospho-PKA substrate (RRXS/T) antibody (rabbit, monoclonal, 100G7E; Cell Signaling Technology #9624), anti-14-3-3 (pan) antibody (rabbit, polyclonal, Cell Signaling Technology #8312), anti-β-tubulin antibody (rabbit, monoclonal, Cell Signaling Technology #2128), anti-GAPDH (D16H11) XP antibody (rabbit, monoclonal, Cell Signaling Technology #5174) and anti-SIK2 antibody (rabbit, polyclonal, Cell Signaling Technology #6919 and Merck #07-1378). Since the anti-SIK2 antibody (Cell Signaling Technology #6919) showed a band similar in size to SIK2 in brains, which was found for both the wild-type and SIK2 KO mice (Fig. S4b), we used the anti-SIK2 antibody (Merck #07-1378) for densitometric analysis of the brain and BAT (Fig. S4c,d, S1). Mice were sacrificed by cervical dislocation under deep anesthesia with sodium pentobarbital (50 mg/kg body weight). Then, BAT and brains were rapidly removed, frozen in liquid nitrogen and stored at −80 °C until use. The BAT and brain were homogenized using a rotor-stator homogenizer (Polytron) in ice-cold lysis buffer (20 mM HEPES pH 7.5, 100 mM NaCl, 10 mM Na_4_P_2_O_7_, 1.5% Triton X 100, 15 mM NaF, 1X PhosSTOP (Roche), 5 mM EDTA, 1X Protease Inhibitor (Roche)), and then centrifuged for 12 min at 14,000 g at 4 °C. For detection of SIK2, brain homogenates were processed for methanol/chloroform precipitation. The pellets were resolved in SDS-PAGE sample buffer. For immunoprecipitation (IP), transiently expressed 3xFLAG-tagged SIK1, SIK2, and SIK3 proteins were extracted with lysis buffer and then centrifuged for 12 min at 14,000 g at 4 °C. The extracted proteins were incubated with a precipitating antibody at 4 °C for 2 h. The beads were washed three times with IP buffer, and the final precipitates were subjected to immunoblotting analysis. The supernatants were separated by SDS–PAGE and transferred to PVDF membranes. The blots were subsequently washed in PBS plus 0.1% Tween-20 (PBS-T) and incubated overnight at 4 °C with primary antibody (1:1000) in PBS-T with 5% bovine serum albumin. The blots were then washed and incubated with horseradish peroxidase-conjugated donkey anti-rabbit IgG (Jackson Immuno Research; 1:5,000 dilution in 5% skim milk and PBS-T). After washing, the blots were exposed to Clarity Western ECL Substrate (Bio-Rad). The chemiluminescence signaling was detected using FUSION Solo 6 S EDGE (Vilber-Lourmat). The membrane for SIK2 was reprobed with an anti-β-tubulin antibody as a loading control. Densitometric analysis of immunoblotting was performed using ImageJ. The area value of SIK2 was divided by that of the loading-controls for each sample. Normalized density values of SIK2 were divided by the mean of the wild-type mice.

### Statistics

Statistical analyses were performed using SPSS Statistics 22 software (IBM) and Prism 8.0 (GraphPad). All data were subjected to the D’Agostino-Pearson (Omnibus K2) normality test for Gaussian distribution and variance. We performed one-way ANOVA followed by Tukey’s test, one-way repeated measures ANOVA, two-way ANOVA, and two-tailed t-test with Bonferroni correction. We performed one-way repeated measures ANOVA for hourly analysis. P < 0.05 was considered statistically significant. Data were presented as mean ± SEM.

## Supplementary information

Supplementary Information.

## Data Availability

All data are available from the corresponding author or available on the Open Science Framework (https://osf.io/upgxm/).
