# Supplementary material for: Loss of the conserved PKA sites of SIK1 and SIK2 increases sleep need
Source: Sci Rep. 2020 May 26;10:8676. doi: 10.1038/s41598-020-65647-0 (PMC7250853; doi:10.1038/s41598-020-65647-0)
Supplement: Supplementary file 1 — Supplementary Information. [file 41598_2020_65647_MOESM1_ESM.pdf]

Supplementary Information

Loss of the conserved PKA sites of SIK1 and SIK2 increases sleep need

Minjeong Park[1], Chika Miyoshi[1], Tomoyuki Fujiyama[1], Miyo Kakizaki[1], Aya Ikkyu[1], Takato Honda[1], Jinhwan Choi[1], Fuyuki Asano[1], Seiya Mizuno[2], Satoru Takahashi[2], Masashi Yanagisawa[1,3,4] and Hiromasa Funato[1,5]

[1]International Institute for Integrative Sleep Medicine (WPI-IIIS), University of Tsukuba, Tsukuba 305-8575, Japan; [2]Laboratory Animal Resource Center, University of Tsukuba, Tsukuba 305-8575, Japan; [3]Department of Molecular Genetics, University of Texas Southwestern Medical Center, Dallas, TX 75390, USA; [4]Life Science Center for Survival Dynamics, Tsukuba Advanced Research Alliance (TARA), University of Tsukuba, Tsukuba, 305-8575 Ibaraki, Japan; [5]Department of Anatomy, Faculty of Medicine, Toho University, Tokyo 143-8540, Japan.

Corresponding authors: Hiromasa Funato and Masashi Yanagisawa
E-mail: yanagisawa.masa.fu@u.tsukuba.ac.jp; funato.hiromasa.km@u.tsukuba.ac.jp

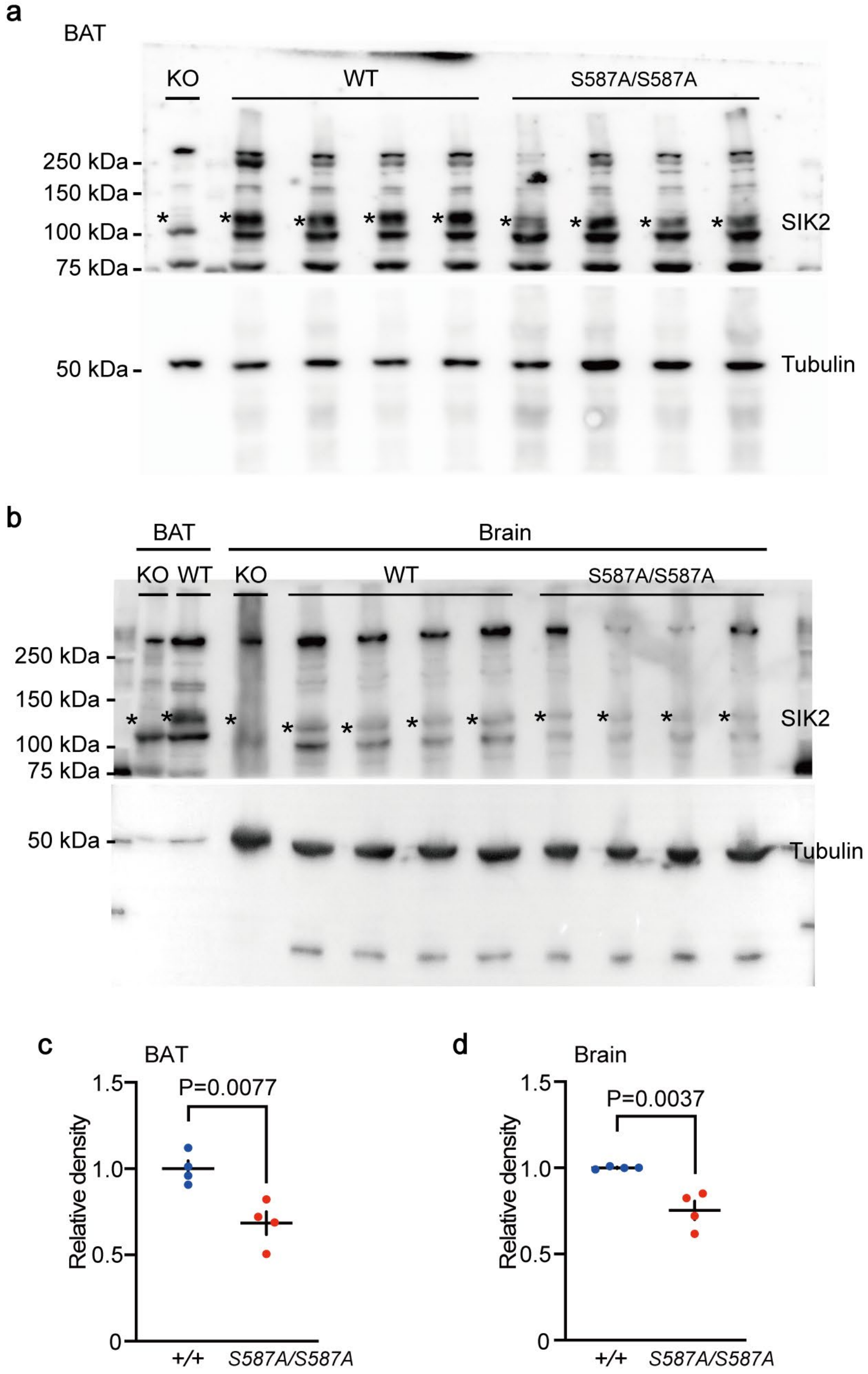

Figure S1 SIK2 protein in BAT and the brain of the *Sik2*$^{S587A/S587A}$ mice.
(**a**) Immunoblot with an anti-SIK2 antibody (Merck #07-1378) of BAT homogenates from the *Sik2*$^{+/+}$ mice (n = 4) and the *Sik2*$^{S587A/S587A}$ mice (n = 4). Asterisks indicate bands corresponding to the SIK2, which was absent in BAT homogenate from the *Sik2*-deficient mice (KO). (**b**) Immunoblot with an anti-SIK2 antibody of brain homogenates from the

Sik2$^{+/+}$ mice (n = 4) and the *Sik2*$^{S587A/S587A}$ mice (n = 4) after methanol/chloroform precipitation. Asterisks indicate bands corresponding to the SIK2, which were absent in BAT and brain homogenates from the *Sik2*-deficient mice (KO). (**c, d**) Densitometric analysis of immunoblotting results (**a**, **b**) of BAT (**c**) and brains (**d**). Two-tailed unpaired T-test. All data are presented as the mean ± SEM.

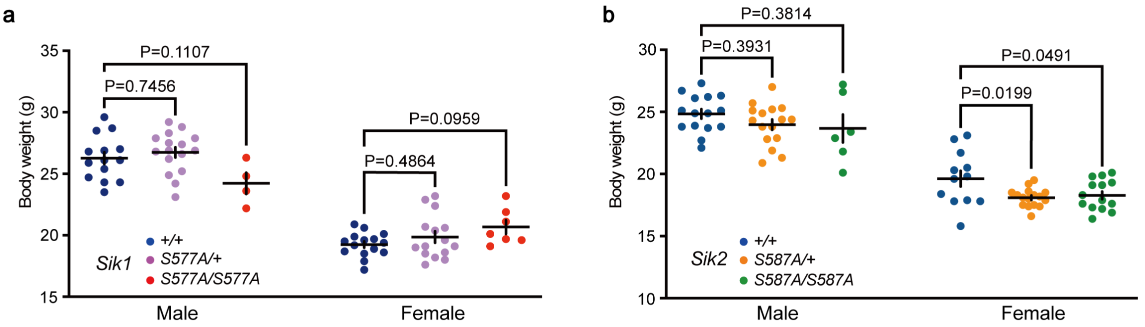

Figure S2 Body weights of the *Sik1*^{S577A} and *Sik2*^{S587A} mice at the age of 8 weeks. (**a**) Body weights of the male and female *Sik1*^{S577A} mice at the age of 8 weeks. *Sik1*$^{+/+}$ mice (male n = 14, female n = 15). *Sik1*$^{S577A/+}$ mice (male n = 16, female n = 15). *Sik1*$^{S577A/S577A}$ mice (male n = 4, female n = 7). (**b**) Body weights of the male and female *Sik2*^{S587A} mice at the age of 8 weeks. *Sik2*$^{+/+}$ mice (male n = 15, female n = 12). *Sik2*$^{S587A/+}$ mice (male n = 16, female n = 16). *Sik2*$^{S587A/S587A}$ mice (male n = 6, female n = 15). One-way analysis of variance followed by Tukey's test. All data are presented as the mean ± SEM.

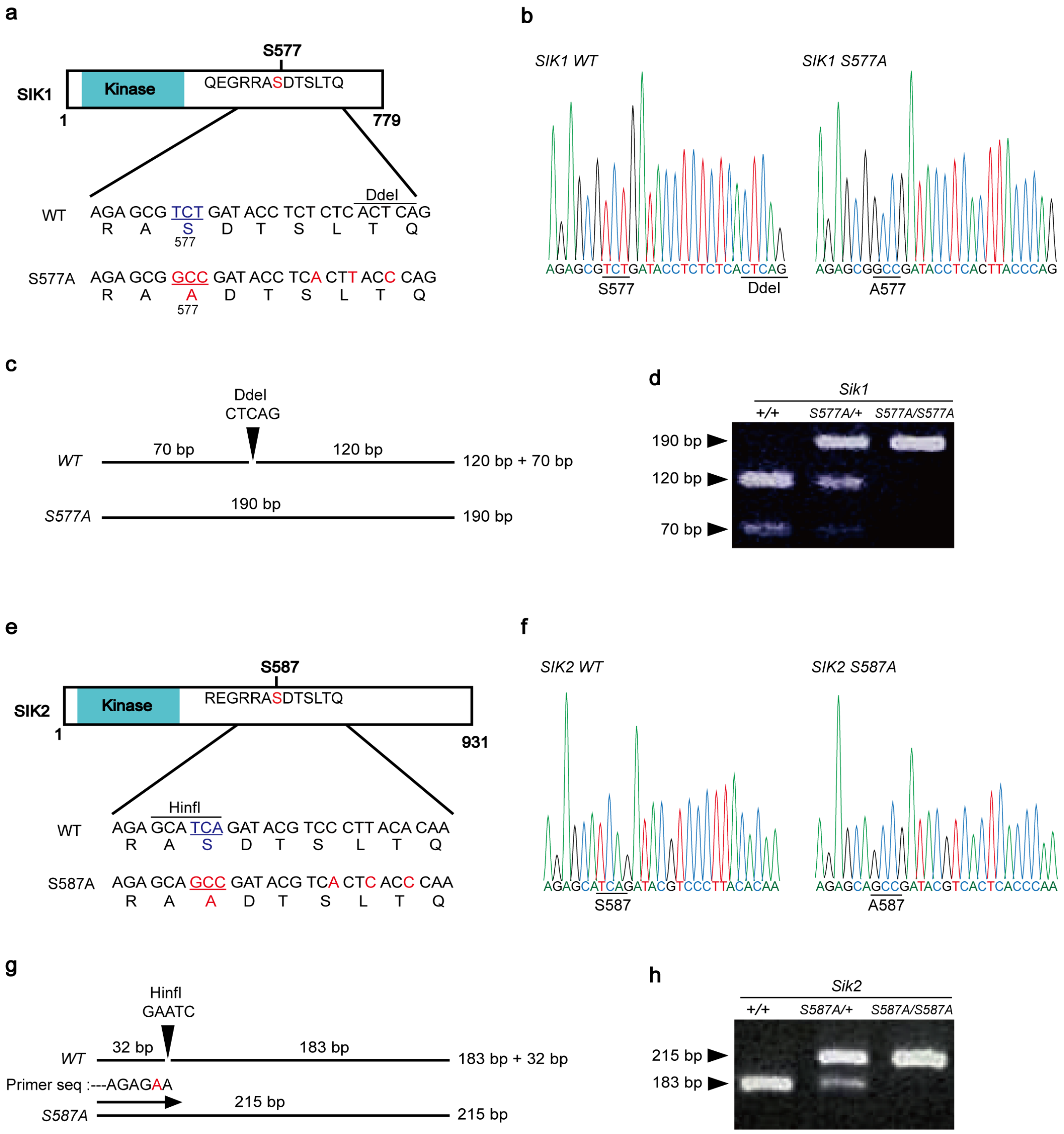

Figure S3 Genotyping of the *Sik1*^{S577A} and *Sik2*^{S587A} mice.

(**a**) The nucleotide and amino acid sequences around SIK1 S577 of the wild-type allele and *S577A* mutant allele. DdeI site exists only in the wild-type allele. (**b**) Direct sequencing of the *Sik1*$^{+/+}$ and *Sik1*$^{S577A/S577A}$ mice. (**c**) DdeI digests a 190-bp PCR product that is derived from the wild-type allele, resulting in 120 bp and 70 bp fragments. (**d**) PCR followed by DdeI digestion determines the genotype of the *Sik1*$^{+/+}$, *Sik1*$^{S577A/+}$, and *Sik1*$^{S577A/S577A}$ mice. (**e**) The nucleotide and amino acid sequences around SIK2 S587 of the wild-type allele and *S587A* mutant allele. HinfI site exists only in the wild-type allele. (**f**) Direct sequencing of the *Sik2*$^{+/+}$ and *Sik2*$^{S587A/S587A}$ mice. (**g**) HinfI digests 215-bp PCR product that is derived from the wild-type allele, resulting in 183 bp and 32 bp fragments. (**h**) PCR followed by HinfI digestion determines the genotype of the *Sik2*$^{+/+}$, *Sik2*$^{S587A/+}$

and *Sik2*$^{S587A/S587A}$ mice.

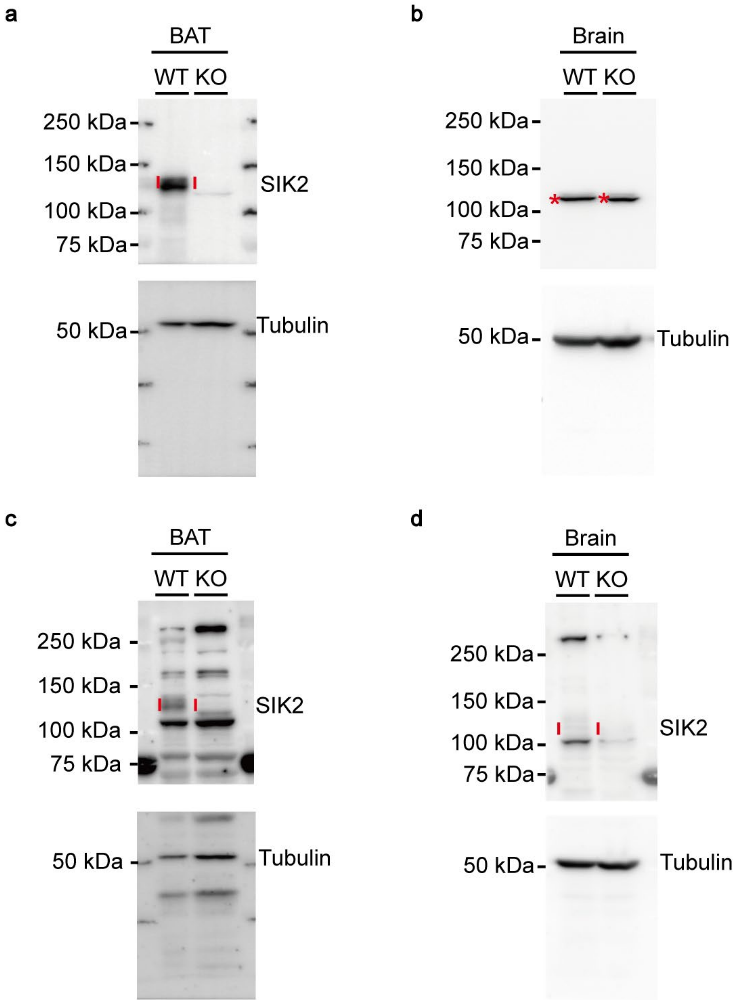

Figure S4 Validation of anti-SIK2 antibodies.

(**a**) An anti-SIK2 antibody (CST #6919) detected SIK2 in BAT of the wild-type (WT) mouse (red bar). The corresponding band (red bar) was absent in BAT of the *Sik2*-deficicent (KO) mice. (**b**) An anti-SIK2 antibody (CST #6919) showed strong bands in brain homogenates of both the WT and KO mice (red asterisks). β-tubulin was used a s loading controls. (**c**) An anti-SIK2 antibody (Merck #07-1378) detected SIK2 in BAT of the WT mouse (red bar). The corresponding band (red bar) was absent in BAT of the KO mice. (**d**) An anti-SIK2 antibody (Merck #07-1378) showed a faint band (red bar) of the same size as SIK2 in the brain of the WT mouse, which was absent in that of the KO mice (red bar).

Figure 1i Input

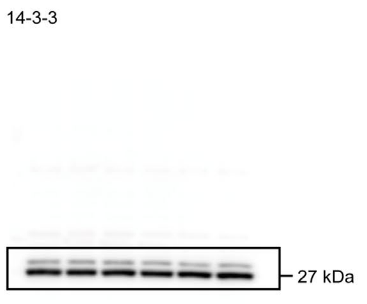

Figure 1i Input

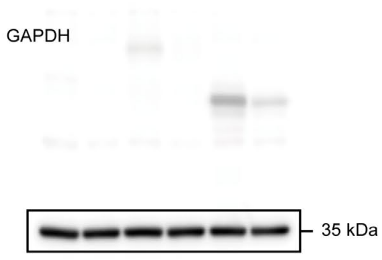

Figure 1i FLAG-IP

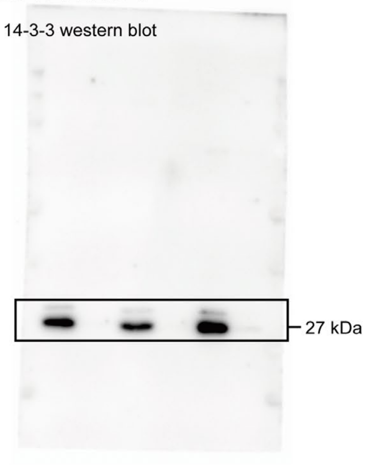

Figure 1i FLAG-IP

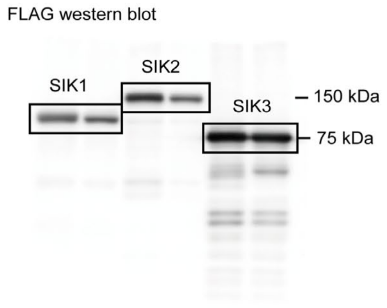

Figure 1i FLAG-IP

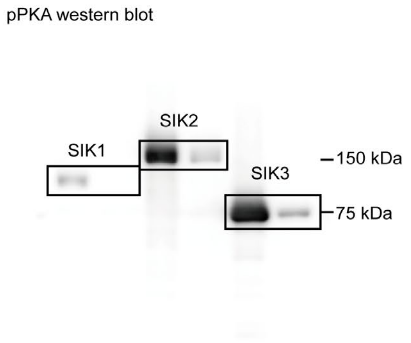

Figure 1j

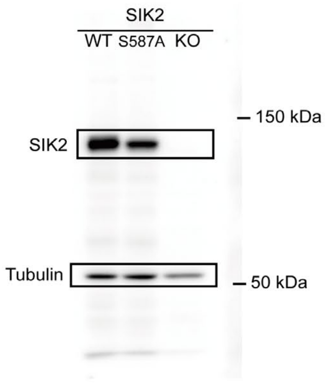

Uncropped blots for figures.

Uncropped blots of Figure 1i and Figure 1j are shown. Black squares represent the images used for figures in the article.
